# Minimally Invasive Myomectomy: A Systematic Review of Techniques, Challenges, and Fertility Outcomes

**DOI:** 10.7759/cureus.85246

**Published:** 2025-06-02

**Authors:** Mohey Aldien Ahmed Elamin Elnour, Ahmed Mohamed Yousif Mohamed, Omer Bilal Mahgoub Ahmed, Mohammed Ahmed Abdelpagee Ismaeil Mohammed Ali, Aliaa Azhari Ahmed Mahmoud, Sarra Ibrahim Abdalla Tabag

**Affiliations:** 1 Neurosurgery, University Hospital Coventry, Coventry, GBR; 2 General Practice, Burjeel Medical City, Abu Dhabi, ARE; 3 General Practice, Al Ahli Medical Care Complex, Al-Ula, SAU; 4 General Surgery, Gurayat General Hospital, Gurayat, SAU; 5 General Practice, Military Hospital, Khartoum, SDN; 6 General Practice, Khartoum Teaching Hospital, Khartoum, SDN

**Keywords:** fertility outcomes, laparoscopic myomectomy, minimally invasive myomectomy, robot-assisted myomectomy, uterine fibroids

## Abstract

Uterine fibroids represent a prevalent gynecological condition with significant implications for fertility and quality of life. This systematic review evaluates the efficacy, safety, and fertility outcomes of minimally invasive myomectomy (MIM) techniques, including laparoscopic myomectomy (LM), robot-assisted laparoscopic myomectomy (RALM), and single-port laparoscopic myomectomy (SPLM), and non-invasive approaches such as ultrasound-guided high-intensity focused ultrasound (USgHIFU). Following Preferred Reporting Items for Systematic Reviews and Meta-Analyses (PRISMA) guidelines, we analyzed 17 studies encompassing diverse surgical methods and reproductive outcomes, with quality assessment performed using the Newcastle-Ottawa scale (NOS). Key findings demonstrate that MIM techniques yield favorable fertility outcomes compared to traditional abdominal myomectomy. Among the included studies, 10 were rated as low risk of bias and 7 as moderate risk on the NOS. Laparoendoscopic single-site myomectomy (LESS-M) and SPLM were associated with higher pregnancy rates (66.7% and 75%, respectively) and shorter time to conception (7.6 vs. 10.1 months for LESS-M vs. conventional LM). RALM showed promise in complex cases, with pregnancy rates of 13-66.7% and no reported uterine ruptures. Non-invasive USgHIFU offered comparable pregnancy rates to LM but was linked to shorter conception times and mixed fetal outcomes, including higher preterm birth rates. Surgical nuances, such as barbed versus nonbarbed sutures, did not significantly impact fertility, while fibroid characteristics and surgeon expertise played pivotal roles. Vaginal birth after LM was deemed safe, with no uterine ruptures reported in large cohorts. However, heterogeneity in study designs, retrospective biases, and limited long-term follow-up data underscore the need for standardized protocols and prospective trials. MIM techniques are a viable option for women seeking fertility preservation, offering reduced morbidity and comparable or superior reproductive outcomes. Future research should prioritize multicenter studies to optimize patient selection and surgical standardization.

## Introduction and background

Uterine fibroids (leiomyomas) represent one of the most prevalent benign gynecological conditions, affecting up to 70-80% of women by age 50, with a disproportionate burden on women of African descent [1]. These hormone-dependent tumors are a leading cause of abnormal uterine bleeding, pelvic pain, and infertility, profoundly impacting quality of life and reproductive autonomy [2]. For women desiring uterine preservation, myomectomy has emerged as the definitive surgical intervention, with minimally invasive techniques, laparoscopic, robotic-assisted, and hysteroscopic approaches, revolutionizing the field by offering reduced morbidity, faster recovery, and improved cosmetic outcomes compared to traditional laparotomy [3,4]. 

The evolution of minimally invasive myomectomy (MIM) reflects advancements in surgical technology and a paradigm shift toward patient-centered care [5]. Laparoscopic myomectomy (LM), first introduced in the 1980s, demonstrated significant advantages, including shorter hospital stays, lower intraoperative blood loss, and decreased postoperative pain [6]. Robotic-assisted systems, such as the da Vinci® platform, further enhanced precision in suturing and tissue dissection, particularly for complex or deep intramural fibroids, albeit with higher costs and longer operative times [7]. Concurrently, hysteroscopic techniques became the gold standard for submucosal fibroids, enabling resection without abdominal incisions [8]. Despite these innovations, challenges persist, including the learning curve for surgeons, risks of intraoperative complications (e.g., hemorrhage, unintended morcellation of occult malignancies), and concerns about fertility implications, such as uterine scar integrity and adhesion formation. 

Fertility outcomes following MIM remain a critical focus, as 20-40% of women with fibroids seek conception post-surgery [9]. Early studies reported conflicting results, with some suggesting increased risks of uterine rupture in laparoscopic cases, while others found no significant differences in live birth rates across surgical approaches. Recent large-scale registries, such as the COMPARE-UF study, have provided robust evidence that pregnancy and live birth rates are comparable between laparoscopic, robotic, and abdominal routes, with success rates ranging from 50% to 80% among women actively attempting conception [10]. However, heterogeneity in study designs, fibroid characteristics (e.g., size, location, FIGO classification), and adjuvant fertility treatments complicates consensus on optimal techniques. For instance, robotic myomectomy has shown promise in managing large or multiple fibroids while maintaining high pregnancy rates, yet concerns about obstetric complications, such as preterm birth and cesarean delivery, warrant further investigation [11].

This systematic review synthesizes contemporary evidence on MIM techniques, procedural challenges, and fertility outcomes to address gaps in clinical guidance. By evaluating comparative efficacy, safety profiles, and long-term reproductive success, this work aims to inform surgical decision-making, enhance patient counseling, and identify priorities for future research in an era where fertility preservation and minimally invasive innovation are inextricably linked.

## Review

Methodology

This systematic review was conducted in accordance with the Preferred Reporting Items for Systematic Reviews and Meta-Analyses (PRISMA) guidelines [12] to ensure methodological rigor, transparency, and reproducibility. The process was structured into distinct phases, including literature search, study selection, data extraction, quality assessment, and data synthesis, as outlined below. To maintain workflow efficiency and adhere to tight research milestones, we opted to proceed without PROSPERO registration during the initial stages.

Eligibility Criteria 

The Population, Intervention, Comparison, Outcomes, and Study Design (PICOS) framework guided eligibility criteria. Studies were included if they evaluated women of reproductive age undergoing MIM (laparoscopic, robotic-assisted, or hysteroscopic) for symptomatic uterine fibroids, with reported outcomes on surgical techniques, intraoperative/postoperative complications, or fertility outcomes (pregnancy rates, live births, obstetric complications). Comparative studies against abdominal myomectomy (AM) or between MIM techniques were prioritized. Exclusion criteria encompassed non-English publications, review articles, editorial letters, case reports, conference abstracts without full data, and studies lacking fertility-specific endpoints. 

Search Strategy and Information Sources

A comprehensive search was executed across four electronic databases (PubMed, Embase, Scopus, and Web of Science) with no geographical restrictions. Search terms were developed in collaboration with a medical librarian and combined Medical Subject Headings (MeSH) with free-text keywords, including permutations of “minimally invasive myomectomy,” “laparoscopic myomectomy,” “robotic myomectomy,” “hysteroscopic myomectomy,” “fertility outcomes,” “pregnancy rate,” and “uterine scar integrity.” To mitigate publication bias, backward and forward citation tracking of included studies and relevant systematic reviews was performed. The complete search for each database is provided in Table 2 in the appendices. 

Study Selection Process

All retrieved records were imported into EndNote 21 (Clarivate Analytics, Philadelphia, PA, USA). Duplicates were automatically removed using EndNote 21 software. Two independent reviewers conducted a two-stage screening process: first, titles and abstracts were assessed against eligibility criteria, followed by full-text evaluation of potentially relevant studies. Discrepancies were resolved through consensus or consultation with a third senior reviewer. The study selection workflow, including reasons for exclusion at the full-text stage, was documented using a PRISMA flow diagram. 

Data Extraction and Management 

A standardized, Microsoft Excel sheet (Microsoft Corporation, Redmond, WA, USA) was utilized to collect variables such as study design, sample size, patient demographics, surgical parameters, complications, and fertility outcomes (time to conception, miscarriage rates, uterine rupture). For randomized controlled trials (RCTs), data on randomization methods and blinding were extracted. Two reviewers independently extracted data, with cross-verification to ensure accuracy. Corresponding authors were contacted for missing or ambiguous data, and studies with incomplete datasets were excluded from quantitative synthesis. 

Quality Assessment

The risk of bias assessment for the 17 included studies was conducted using the Newcastle-Ottawa scale (NOS) [13], which evaluates studies based on three domains: Selection (maximum 4 stars), Comparability (maximum 2 stars), and Outcome (maximum 3 stars). Each study was assigned a total score ranging from 0 to 9, with higher scores indicating lower risk of bias. The overall risk of bias was categorized as "Low" (scores ≥7), "Moderate" (scores 5-6), or "High" (scores ≤4), consistent with established NOS guidelines.

Data Synthesis

Given the substantial heterogeneity in surgical techniques, outcome measures, and study designs across the included literature, a meta-analysis was deemed inappropriate. Instead, a comprehensive narrative synthesis was conducted to analyze and interpret the findings thematically. This approach allowed for a nuanced exploration of the relationships between MIM techniques, procedural challenges, and fertility outcomes while accounting for variations in study populations, fibroid characteristics, and methodological quality.

Results

Studies' Selection Process

**Figure 1:**
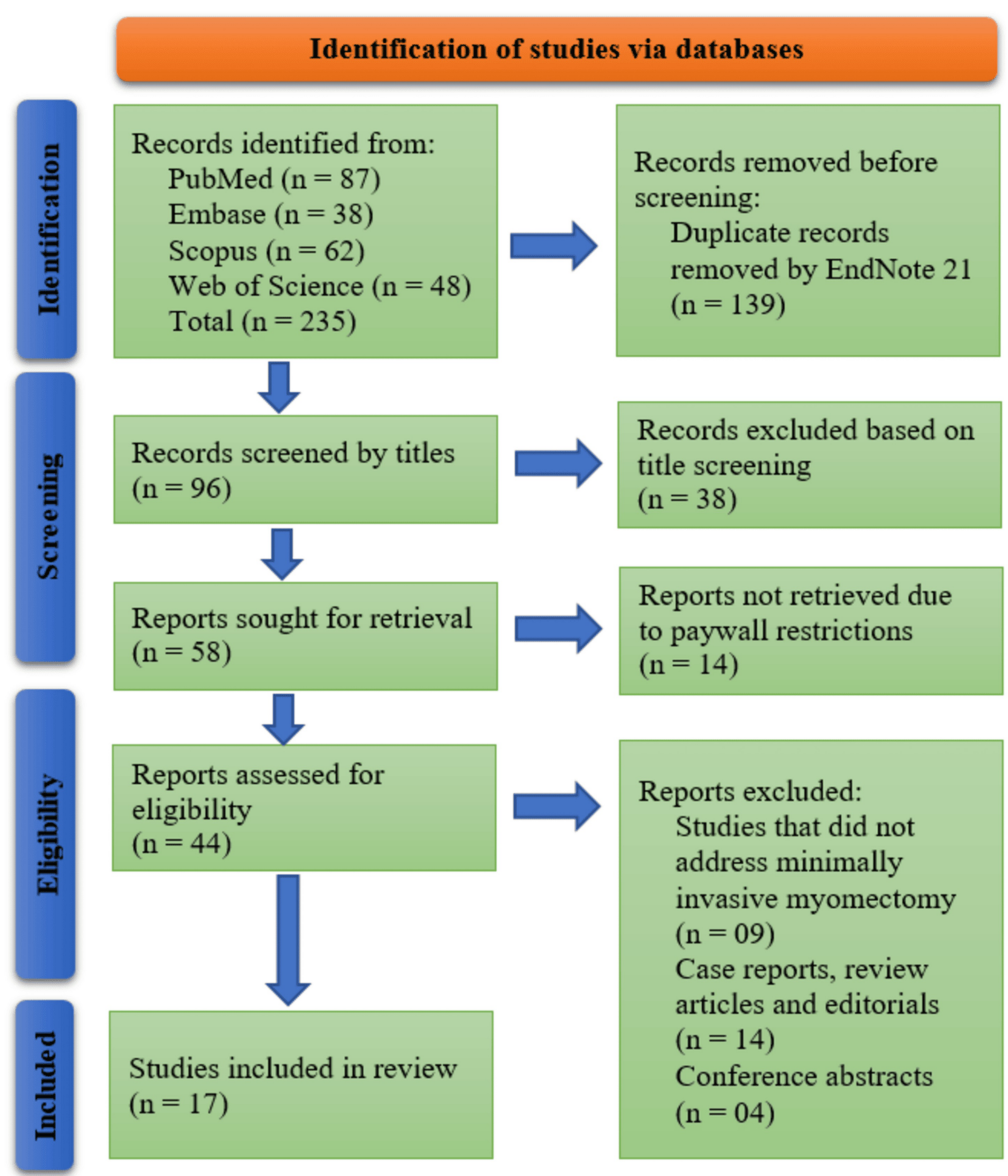
PRISMA Flow Diagram Illustrating the Study Selection Process PRISMA: Preferred Reporting Items for Systematic Reviews and Meta-Analyses

A total of 235 records were identified through database searches: PubMed (n = 87), Embase (n = 38), Scopus (n = 62), and Web of Science (n = 48). After the removal of 139 duplicate records using EndNote 21, 96 records remained for title screening. Following this, 38 records were excluded based on titles. Of the remaining 58 reports sought for retrieval, 14 could not be retrieved due to paywall restrictions. The 44 accessible reports were assessed for eligibility, among which 27 were excluded for the following reasons: studies not addressing MIM (n = 9), case reports, review articles, and editorials (n = 14), and conference abstracts (n = 4). Ultimately, 17 studies were included in the systematic review (Figure 1).

Study Characteristics and Surgical Approaches

A total of 17 studies [14-30] were included in this review, encompassing a variety of study designs such as retrospective cohort studies, prospective clinical studies, and matched case-control trials. Surgical techniques examined included LM, single-port laparoscopic myomectomy (SPLM), robot-assisted laparoscopic myomectomy (RALM), AM, and non-invasive ultrasound-guided high-intensity focused ultrasound (USgHIFU). Across all studies, LM was the most commonly evaluated technique either independently or in comparison with others (Table 1).

**Table 1:** Summary of Included Studies LESS-M, Laparoendoscopic Single-Site Myomectomy; LM, Laparoscopic Myomectomy; SPLM, Single-Port Laparoscopic Myomectomy; RALM, Robot-Assisted Laparoscopic Myomectomy; AM, Abdominal Myomectomy; USgHIFU, Ultrasound-guided High-Intensity Focused Ultrasound; TAM, Transabdominal Myomectomy; LIM, Laparoscopic Intracapsular Myomectomy; LAIM, Laparoscopically Assisted Intracapsular Myomectomy; LSM, Laparoscopic Myomectomy; FIGO, International Federation of Gynecology and Obstetrics; CS, Cesarean Section

Author(s)	Country	Study Design	Sample Size	Patient Population	Type of Myomectomy	Surgical Approach	Mean Age (years)	Fibroid Size / Number	Follow-up Duration	Fertility Outcome Reported	Key Findings
Kim et al., [14] 2014	South Korea	Prospective matched case-control	135 (45 LESS-M, 90 LM)	Women with symptomatic myomas	LESS-M and Conventional LM	Laparoscopic (Single-site vs Multi-port)	Not specified	Not specified	24.4 vs 23.2 months	Pregnancy rate: 66.7% (LESS-M) vs 50.0% (LM); Full-term delivery: 66.7% vs 58.3%; Time to first pregnancy: 7.6 vs 10.1 months	LESS-M is feasible, safe and has comparable obstetric outcomes to conventional LM
Hong et al., [15] 2021	South Korea	Single-center retrospective study	504 procedures (502 patients)	Women undergoing SPLM for symptom relief or growing myomas	Single-port laparoscopic myomectomy (SPLM)	Single-port laparoscopy	40.6 ± 6.6	Avg. 2.3 ± 2.2 myomas; Largest 6.8 ± 2.4 cm	Surgery-to-pregnancy interval: 15.6 ± 12.2 months	Yes	SPLM showed good operative and obstetric outcomes, with 75% pregnancy rate and 69.6% live birth rate among those who attempted conception.
Cela et al., [16] 2013	Italy	Retrospective observational	48	Women undergoing RALM between 2007 and 2011	Robot-assisted laparoscopic myomectomy (RALM)	Laparoscopic (robot-assisted)	35	Not reported	6 months (for endocrine follow-up), pregnancy outcomes followed long-term	Yes – 13% pregnancy rate, 8 pregnancies, 7 deliveries (6 C-sections, 1 vaginal), no spontaneous abortions or uterine ruptures	RALM has favorable reproductive outcomes and does not impair ovarian function
Pitter et al., [17] 2015	United States	Retrospective Survey	426	Women undergoing RALM for fibroids, symptom relief, or infertility	Robot-assisted laparoscopic myomectomy (RALM)	Laparoscopic (robot-assisted)	Not reported	Not reported	>3 years	Yes – 66.7% achieved pregnancy after >3 years if infertility was indication and symptom-free	RALM showed sustained symptom relief (70% symptom-free); improved fertility in symptom-free patients; symptom recurrence linked to multiple factors (e.g., dyspareunia, diabetes); fertility positively associated with prior pregnancy, Caucasian race; negatively associated with age and symptom recurrence
Flyckt et al., [18] 2016	United State	Retrospective Cohort	134	Women aged 18–39 undergoing myomectomy	Robotic-assisted, laparoscopic, abdominal	Robotic-assisted (n=25), laparoscopic (n=28), open/abdominal (n=81)	Not specified	Not reported	Median 8 years	Yes (Spontaneous pregnancy rate)	No significant difference in long-term bleeding or fertility outcomes among surgical groups
Fukuda et al., [19] 2013	Japan	Retrospective cohort	105	Women who delivered after myomectomy (2004–2012)	Laparoscopic and Abdominal	LM vs. AM (unspecified access routes)	Not reported	Not reported	Not specified; patients followed until delivery	Yes (perinatal and delivery outcomes)	No significant differences in perinatal outcomes between LM and AM; high rates of successful vaginal delivery in both groups
Kumakiri et al., [20] 2008	Japan	Prospective Clinical Study (Canadian Task Force II-2)	1334 (LM); 221 (became pregnant); 111 (followed up for delivery)	Women undergoing LM from Jan 2000 to Dec 2005	Laparoscopic Myomectomy	Laparoscopic	Not reported	Mean fibroid diameter: 66.1 ± 18.8 mm; Mean number: 3.5 ± 3.6	Follow-up through Dec 2006	Yes – 59/74 had successful vaginal delivery post-LM	Vaginal birth after LM is feasible and safe in selected patients; no uterine rupture reported
Wu et al., [21] 2020	China	Comparative Retrospective Study	676 (320 USgHIFU, 336 LM)	Women with symptomatic fibroids desiring pregnancy	Laparoscopic Myomectomy (LM), USgHIFU (non-invasive)	Laparoscopic, Non-invasive (USgHIFU)	Not specified	Not specified	Median 5 years (range 1–8)	Yes (Pregnancy rate, delivery outcomes, time to conception)	Pregnancy rates similar; USgHIFU shortened time to pregnancy; USgHIFU had lower cesarean and placental complication rates, but higher preterm birth and fetal complications; risk of uterine rupture in both groups
Ordás et al., [22] 2022	Spain	Retrospective cohort	254	Women undergoing myomectomy	Laparoscopic myomectomy (LM), abdominal myomectomy (AM)	Laparoscopic, Abdominal	Not reported	LM: 1.8 ± 1.5 fibroids, 7.6 ± 2.7 cmAM: 3 ± 2.9 fibroids, 10.2 ± 5.4 cm	Not reported	Yes – LM: 30.8% vs AM: 16.8% pregnancy rate; 69% vaginal delivery rate; no uterine rupture	LM associated with shorter hospitalization, longer surgical time, fewer and smaller fibroids removed, higher pregnancy rate, no difference in complication rates
Arena et al., [23] 2021	Italy	Retrospective monocentric cohort study (with prospective pregnancy follow-up for some)	164 (83 nonbarbed, 81 barbed)	Women >18 years undergoing laparoscopic myomectomy and seeking pregnancy	Laparoscopic myomectomy	Laparoscopic	Not specified	FIGO types 3, 4, 5, 6 (number/size not stated)	Not specified	Yes – pregnancy rates, delivery modes, complications	Barbed sutures showed similar reproductive outcomes and complication rates compared to smooth sutures
Tian et al., [24] 2015	China	Retrospective and Prospective Cohort	268 (157 retrospective, 111 prospective)	Women with fertility requirements post-myomectomy	Transabdominal myomectomy (TAM), laparoscopic myomectomy (LM)	Transabdominal and Laparoscopic	Not specified	Comparable characteristics between groups	Not specified	Yes – cumulative pregnancy rates and obstetric outcomes assessed	LM had faster recovery, less analgesia use, shorter ileus and ambulation time; similar pregnancy rates; higher uterine scar defect in the LM group; longer contraception interval and higher cesarean rate due to myomectomy in the TAM group
Paul et al., [25] 2022	India	Monocentric retrospective cohort study with prospective follow-up	343 included; 235 responded (Group A: 120, Group B: 115)	Women undergoing laparoscopic myomectomy between 2004 and 2017	Laparoscopic myomectomy	Laparoscopic	Not reported	Not reported	April–May 2020 (13–16 years from first surgeries)	Yes (pregnancy rate, live births, miscarriages, ectopic pregnancies)	No significant difference in reproductive outcomes between barbed and conventional sutures; both approaches safe and effective
Prapas et al., [26] 2020	Greece	Prospective observational	273	Reproductive-age women with intramural or subserous myomas (FIGO type 3–6, 4–14 cm)	Intracapsular single-layer myomectomy	Laparoscopic (LIM) and Laparoscopically assisted (LAIM)	Not reported	4–14 cm fibroids	3–36 months (for fertility outcomes)	Yes (121 pregnancies reported, no uterine ruptures)	Safe and effective; reduced uterine incision size; no severe complications; successful fertility outcomes
Koo et al., [27] 2015	Canada	Retrospective cohort study	523	Women who underwent laparoscopic myomectomy (LSM) at the same center between 1994 and 2012, and had follow-up through pregnancy	Laparoscopic Myomectomy (LSM)	Laparoscopic	Not specified	Multiple myomas removed in 35.2% of cases, mean myoma diameter 4.9 cm	14 months (mean interval between surgery and pregnancy)	Yes (400 full-term deliveries, 100 vaginal deliveries)	LSM is safe for women of reproductive age who wish to become pregnant. Uterine rupture is rare (0.6%), regardless of myoma features. Further studies are needed.
Bernardi et al., [28] 2014	Germany	Retrospective observational	59	Women aged 23–42 with the desire to have children and symptomatic uterine leiomyoma	Laparoscopic myomectomy	Laparoscopic	23–42	Not specified	73.55 months	Yes	LM reduced the abortion rate, increased the CS rate, and affected uterine rupture risk.
Huberlant et al., [29] 2020	France	Retrospective cohort	53	Women with symptomatic leiomyomas desiring pregnancy	Robot-assisted laparoscopic myomectomy (RALM)	Laparoscopic (robot-assisted)	Not provided	Mean number: 2 ± 1.5, Mean size: 69 ± 17.7 mm	3 months post-surgery (office hysteroscopy)	Clinical pregnancy rate: 52.8%, Live birth rate: 41.5%	Over half of the patients became pregnant. Low rate of obstetrical complications, no uterine ruptures.
Kim et al., [30] 2013	South Korea	Retrospective Study	415 women	Women with uterine leiomyomas	Laparoscopic and laparotomic myomectomy	Laparoscopic, Laparotomic	Not provided	Not provided	26.5 months (laparoscopic), 23.9 months (laparotomic)	Yes	Similar obstetric outcomes between groups, with complications in the laparoscopic group (neonatal death, postpartum hemorrhage, uterine dehiscence). 3.7% risk of uterine rupture or dehiscence after laparoscopic myomectomy.

Fertility and Pregnancy Outcomes

Fertility outcomes varied among surgical techniques. Kim et al. [14] demonstrated that laparoendoscopic single-site myomectomy (LESS-M) achieved a 66.7% pregnancy rate compared to 50.0% in conventional LM, with comparable full-term delivery rates (66.7% vs. 58.3%) and shorter time to pregnancy (7.6 vs. 10.1 months). Hong et al. [15] reported high fertility success for SPLM with a 75% pregnancy rate and 69.6% live birth rate among women who attempted conception. Robot-assisted techniques also yielded favorable results: Cela et al. [16] found a 13% pregnancy rate with no uterine ruptures or spontaneous abortions post-RALM, while Pitter et al. [17] observed a 66.7% pregnancy rate among symptom-free women following RALM, especially in those with prior pregnancy and younger age.

Flyckt et al. [18] compared robotic-assisted, laparoscopic, and open myomectomies and reported no significant difference in long-term fertility outcomes. Similarly, Fukuda et al. [19] found no differences in perinatal outcomes between LM and AM, with high rates of successful vaginal deliveries in both groups. Kumakiri et al. [20], in a large prospective study, reported that 59 out of 74 women who conceived post-LM had successful vaginal deliveries with no uterine rupture, affirming the safety of vaginal birth after LM.

Wu et al. [21] conducted a comparative study between LM and USgHIFU and found similar pregnancy rates, though USgHIFU resulted in shorter time to conception and lower cesarean rates but increased preterm birth and fetal complications. Ordás et al. [22] showed that LM was associated with a significantly higher pregnancy rate than AM (30.8% vs. 16.8%) and a high vaginal delivery rate (69%), with no reported uterine rupture.

Surgical Techniques and Fertility Considerations

Arena et al. [23] examined the influence of suture type in LM, reporting no significant difference in fertility or complication rates between barbed and smooth sutures. Tian et al. [24] compared TAM and LM, highlighting LM’s advantages in postoperative recovery and pain management. Although fertility outcomes were similar between the two groups, LM showed a higher incidence of uterine scar defects, while TAM was associated with higher cesarean delivery rates [25,29,30].

Quality Assessment

**Figure 2:**
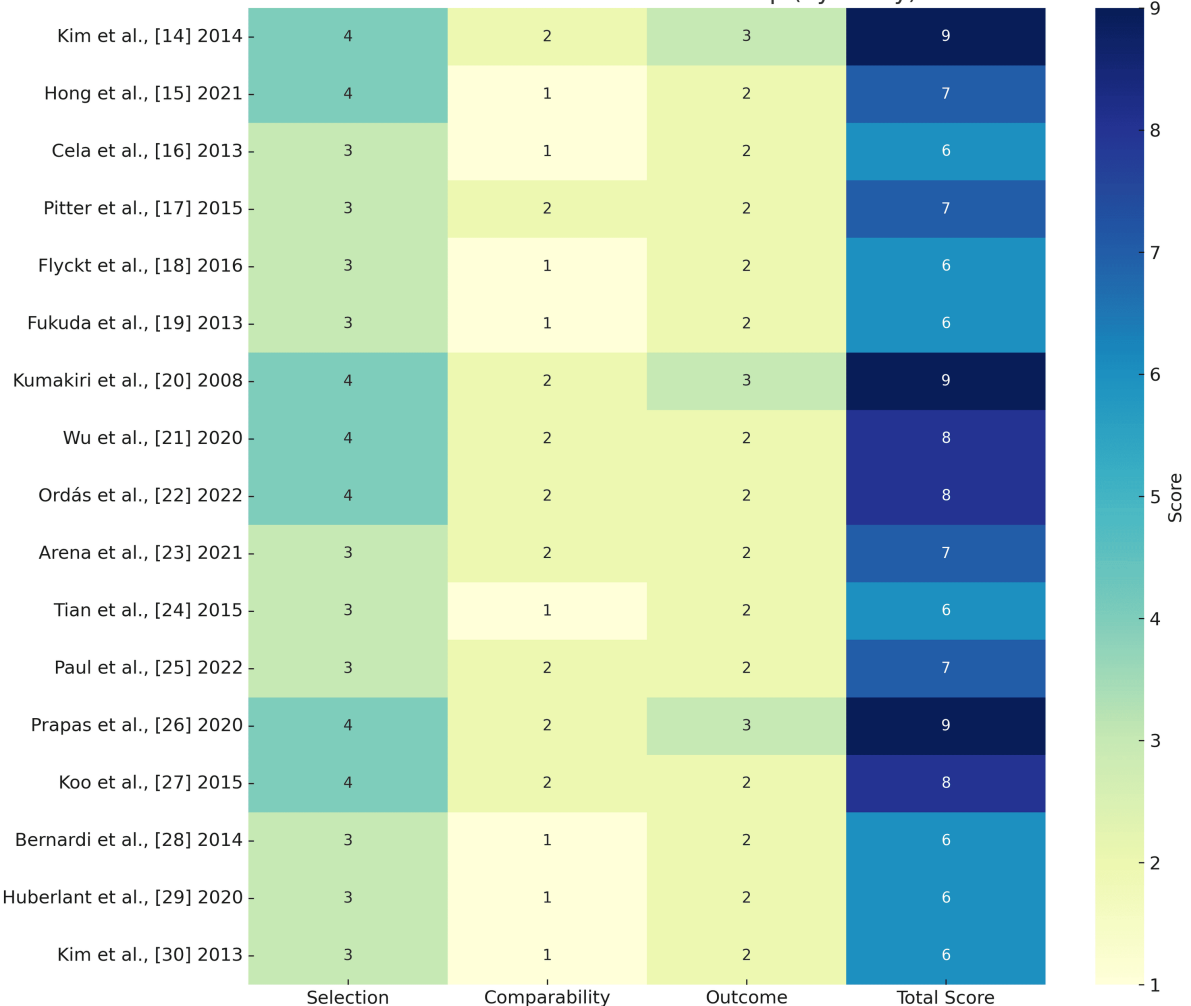
Quality Assessment Using the NOS Tool Selection (max 4), Comparability (max 2), and Outcome (max 3). The Total Score (max 9) determines the risk of bias. 7–9: Low risk (dark blue); 5–6: Moderate risk (light green); 1-4: High risk (light yellow) NOS: Newcastle-Ottawa scale

The risk of bias assessment revealed that 10 studies were rated as low risk [14,15,17,20-23,25-27], while seven studies were classified as moderate risk [16,18,19,24,28-30]. No studies were deemed high risk. The low-risk studies consistently scored well across all domains, particularly in Selection and Comparability, with some achieving the maximum score of 9 [14,20,26]. In contrast, the moderate-risk studies often lost points in Comparability or Outcome, primarily due to insufficient control for confounding factors or limitations in outcome assessment. These findings indicate that while the majority of studies were methodologically sound, a notable proportion had moderate biases that should be considered when interpreting the results (Figure 2).

Discussion

Overview of MIM and Fertility Outcomes

The findings of this systematic review highlight the evolving landscape of MIM techniques and their implications for fertility outcomes. The included studies encompass a diverse range of approaches, including LM, RALM, SPLM, and non-invasive methods like USgHIFU. Collectively, these studies demonstrate that MIM is not only feasible and safe but also associated with favorable fertility outcomes, albeit with variations depending on the surgical approach, patient characteristics, and fibroid features.

Comparison of Techniques

One of the key observations from this review is the comparable fertility outcomes between conventional LM and newer techniques such as LESS-M and SPLM. Kim et al. [14] reported a higher pregnancy rate (66.7%) and shorter time to conception (7.6 months) with LESS-M compared to conventional LM (50.0%, 10.1 months), indicating a potential reproductive benefit of single-site approaches; however, further high-quality studies are needed to confirm these findings. Similarly, Hong et al. [15] found that SPLM yielded a 75% pregnancy rate and a 69.6% live birth rate among women attempting conception, further supporting the reproductive efficacy of minimally invasive techniques. These results align with existing literature that emphasizes the benefits of reduced surgical trauma and faster recovery in single-port and single-site procedures, which may contribute to improved fertility outcomes [31]. However, it is noteworthy that these studies were conducted in homogeneous populations (e.g., South Korea), which may limit the generalizability of the findings to other demographic groups.

RALM has also emerged as a promising technique, particularly for complex cases requiring meticulous suturing. Cela et al. [16] reported a 13% pregnancy rate and no cases of uterine rupture or spontaneous abortion post-RALM, underscoring its safety and potential to preserve reproductive function. Pitter et al. [17] further reinforced these findings, noting that 66.7% of symptom-free patients achieved pregnancy within three years post-RALM. The study also identified factors such as age, symptom recurrence, and prior pregnancy history as significant predictors of fertility outcomes, which is consistent with broader literature on fibroid management [32]. Flyckt et al. [18], however, found no significant differences in long-term fertility outcomes between robotic, laparoscopic, and abdominal myomectomy, suggesting that the choice of technique may depend on surgeon expertise and patient-specific factors rather than inherent superiority of one method over another. This dichotomy in findings highlights the need for larger, multicenter studies to clarify the role of RALM in fertility preservation.

The comparison between laparoscopic and AM revealed nuanced differences in obstetric outcomes. Fukuda et al. [19] and Ordás et al. [22] both reported no significant differences in perinatal outcomes between LM and AM, but Koo et al. [27] noted a higher pregnancy rate (30.8% vs. 16.8%) and shorter hospitalization in the LM group. These findings are consistent with meta-analyses by Yang et al. [33], which concluded that LM is associated with fewer adhesions and quicker recovery, potentially enhancing fertility. However, Tian et al. [24] observed a higher incidence of uterine scar defects in the LM group, raising concerns about uterine integrity during subsequent pregnancies. This aligns with the study by Chervenak et al. [34], which cautions that the depth of fibroid excision and suturing techniques in LM may influence uterine rupture risk. Kumakiri et al. [20] and Koo et al. [27] provided reassuring data on vaginal birth after LM, with no uterine ruptures reported in large cohorts, suggesting that LM is safe for women desiring future pregnancy, provided appropriate surgical protocols are followed.

Non-invasive techniques like USgHIFU present an intriguing alternative for women seeking fertility preservation. Wu et al. [21] found comparable pregnancy rates between USgHIFU and LM but noted a shorter time to conception and lower cesarean rates in the USgHIFU group. However, the higher preterm birth and fetal complication rates in the USgHIFU group warrant caution, as these findings contradict earlier studies by Liu et al. [35], which reported minimal adverse effects. The variability in outcomes may stem from differences in fibroid characteristics or operator experience, emphasizing the need for standardized protocols in USgHIFU applications.

Suture Methods

Surgical nuances, such as suturing techniques, also play a critical role in fertility outcomes. Arena et al. [23] and Paul et al. [25] compared barbed versus nonbarbed sutures in LM and found no significant differences in pregnancy rates or complications. This challenges the notion that barbed sutures, which are easier to handle, might compromise uterine healing. Prapas et al. [26] further demonstrated that single-layer suturing in intracapsular myomectomy is sufficient for normal wound healing, with no uterine ruptures reported in their cohort. These findings are supported by the experimental study by Kim et al. [36], which advocates for minimalistic suturing to reduce ischemia and adhesion formation. However, Bernardi et al. [28] reported an increased cesarean rate post-LM, attributing it to concerns about uterine integrity, suggesting that surgeon perception and institutional policies may influence obstetric management independently of actual surgical outcomes.

Fibroid Characteristics

The impact of fibroid characteristics on fertility outcomes remains a critical consideration. While many included studies did not systematically analyze fibroid size or number, Kumakiri et al. [20] and Koo et al. [27] noted that multiple myomas or larger fibroids did not significantly increase uterine rupture risk. This contrasts with older studies by Neves-e-Castro [37], which associated larger fibroids with higher complication rates. The discrepancy may reflect advances in surgical techniques or patient selection criteria, underscoring the importance of individualized treatment planning. Huberlant et al. [29] and Kim et al. [30] also highlighted the low rate of obstetrical complications post-MIM, with uterine rupture rates below 4%, reinforcing the safety of these techniques in well-selected patients.

Research Gaps and Future Directions

Despite the promising findings, this review reveals several gaps in the literature. Many studies lacked long-term follow-up, focusing primarily on short-term pregnancy rates rather than live birth or neonatal outcomes. Additionally, heterogeneity in outcome reporting (e.g., varying definitions of "fertility success") complicates cross-study comparisons. The predominance of single-center retrospective studies, as seen in the studies by Flyckt et al. [18] and Pitter et al. [17], introduces potential biases, such as selection bias and unmeasured confounding. Prospective RCTs are needed to establish causal relationships between surgical techniques and fertility outcomes. Furthermore, the underrepresentation of diverse populations in studies like those by Kim et al. [14] and Hong et al. [15] limits the generalizability of the results to global populations.

Limitations

This systematic review has several limitations. First, the included studies exhibit significant heterogeneity in design, patient populations, and outcome measures, precluding meta-analysis. Second, the predominance of retrospective and observational studies introduces potential biases, such as recall and selection bias. Third, many studies lacked detailed reporting of fibroid characteristics (e.g., size, location), which are critical determinants of fertility outcomes. Fourth, the follow-up durations varied widely, with some studies reporting outcomes only up to a few months post-surgery, limiting insights into long-term fertility and obstetric risks. Finally, the exclusion of non-English studies may have omitted relevant data from other regions.

## Conclusions

Laparoscopic, robot-assisted, and single-port approaches demonstrate comparable or superior reproductive outcomes relative to traditional AM, with low rates of complications such as uterine rupture. Non-invasive methods like USgHIFU offer promising alternatives but require further investigation to address inconsistencies in fetal outcomes. Surgical nuances, including suturing techniques and fibroid characteristics, play pivotal roles in shaping fertility outcomes, highlighting the need for individualized surgical planning. However, the current evidence is limited by heterogeneous study designs and short follow-up periods. Future research should prioritize prospective, multicenter studies with standardized outcome measures to refine patient selection criteria and optimize surgical protocols. Until then, MIM remains a cornerstone in the management of fibroids for women desiring future pregnancy.

## Appendices

**Table 2:** Search Strategy

Database	Search Terms	Filters/Restrictions
PubMed	("Myomectomy"[MeSH] OR "fibroid removal" OR "uterine fibroid surgery" OR "leiomyoma surgery") AND ("Minimally Invasive Surgical Procedures"[MeSH] OR laparoscopic OR robotic OR hysteroscopic) AND (fertility OR "reproductive outcome" OR conception OR pregnancy)	Language: English, Humans Publication Date: 2000–2025
Embase	('myomectomy'/exp OR 'fibroid removal' OR 'leiomyoma surgery' OR 'uterine fibroid surgery') AND ('minimally invasive surgery'/exp OR laparoscopic OR robotic OR hysteroscopic) AND (fertility OR 'reproductive outcome' OR conception OR pregnancy)	Language: English, Humans Years: 2000–2025
Scopus	TITLE-ABS-KEY (myomectomy OR "fibroid removal" OR "leiomyoma surgery" OR "uterine fibroid surgery") AND TITLE-ABS-KEY ("minimally invasive" OR laparoscopic OR robotic OR hysteroscopic) AND TITLE-ABS-KEY (fertility OR "reproductive outcome" OR conception OR pregnancy)	Language: English, Document Type: Article Years: 2000–2025
Web of Science	TS=(myomectomy OR "fibroid removal" OR "leiomyoma surgery" OR "uterine fibroid surgery") AND TS=("minimally invasive" OR laparoscopic OR robotic OR hysteroscopic) AND TS=(fertility OR "reproductive outcome" OR conception OR pregnancy)	Language: English, Document Type: Article Timespan: 2000–2025
